# An integrated framework for the geographic surveillance of chronic disease

**DOI:** 10.1186/1476-072X-8-69

**Published:** 2009-11-30

**Authors:** Nikolaos Yiannakoulias, Lawrence W Svenson, Donald P Schopflocher

**Affiliations:** 1 School of Geography and Earth Sciences, 1280 Main Street West, McMaster University, Hamilton, Ontario, L8S4K1, Canada; 2 Public Health Division, Alberta Health and Wellness, PO Box 1360 Station Main, Edmonton, Alberta, T5J2N3, Canada; 3 Faculty of Nursing, 3rd Floor Clinical Sciences Building, University of Alberta, Edmonton, Alberta, T6G2G3, Canada

## Abstract

**Background:**

Geographic public health surveillance is concerned with describing and disseminating geographic information about disease and other measures of health to policy makers and the public. While methodological developments in the geographical analysis of disease are numerous, few have been integrated into a framework that also considers the effects of case ascertainment bias on the effectiveness of chronic disease surveillance.

**Results:**

We present a framework for the geographic surveillance of chronic disease that integrates methodological developments in the spatial statistical analysis and case ascertainment. The framework uses an hierarchical approach to organize and model health information derived from an administrative health data system, and importantly, supports the detection and analysis of case ascertainment bias in geographic data. We test the framework on asthmatic data from Alberta, Canada. We observe high prevalence in south-western Alberta, particularly among Aboriginal females. We also observe that persons likely mistaken for asthmatics tend to be distributed in a pattern similar to asthmatics, suggesting that there may be an underlying social vulnerability to a variety of respiratory illnesses, or the presence of a diagnostic practice style effect. Finally, we note that clustering of asthmatics tends to occur at small geographic scales, while clustering of persons mistaken for asthmatics tends to occur at larger geographic scales.

**Conclusion:**

Routine and ongoing geographic surveillance of chronic diseases is critical to developing an understanding of underlying epidemiology, and is critical to informing policy makers and the public about the health of the population.

## Background

Modern public health surveillance involves the routine collection, analysis, synthesis, and timely dissemination of health information, including infectious, non-infectious, acute and chronic diseases, mortality and other indicators of health [1-3]. Geographic public health surveillance is concerned with analyzing and disseminating geographic information as part of the general practice of public health surveillance, and comprises a mixture of fields, including geographic information systems (GIS), spatial decision support, epidemiology and biostatistics [4]. Geographic public health surveillance activities can help identify environmental or social hazards, geographic concentrations of at-risk groups, shortages of treatment and preventative resources and variations in the way illness is understood in the population and the medical community. Routine monitoring of geographic patterns of health ensures that policy makers and the public are aware of shifts in epidemiology, demography and treatment, and facilitates long term planning of health care and resource allocation. Routine and ongoing geographic public health surveillance is also critical for understanding changes in the geographies of health, especially since cross-sectional research provides less insight into changes in the underlying patterns of health and disease.

The practice of public health surveillance encounters a number of important challenges. Concerns about privacy and the authority of public health officials force surveillance practice to balance the rights of the individual with the collective responsibility of maintaining public welfare. There are also methodological challenges in public health surveillance, many of which are either distinctly geographical in nature, or are influenced by geographical realities. The four we very briefly discuss below are the small numbers problem, the multiple comparisons problem, the modifiable areal units problem and case ascertainment bias.

### Small numbers problem

For many diseases, small stochastic differences in the number of cases from place to place can result in large apparent but statistically unimportant differences in disease risk. Maps of absolute and relative risk can show considerable apparent variability even when underlying risk is constant. This problem is of particular concern when the underlying populations differ from place to place; in these instances, places have different variance characteristics, which make direct comparisons of rates even more challenging. Many solutions have been considered to deal with the small numbers problem in disease mapping generally, including non-parametric methods [5-7], multiple membership multilevel models [8,9] and Bayesian approaches [10-12].

### Multiple comparisons problem

Maps can provide misleading information even in the absence of statistical uncertainty. Maps of absolute and relative risk often encourage, at least implicitly, comparisons of values--such as the observation that risk in one region is higher or lower than risk in another region. These comparisons amount to ad hoc hypothesis tests in which apparent differences may not represent clinical or statistically significant differences when considered in the context of the large number of possible comparisons that could be made. Such comparisons can lead to unjustified alarm or concern, and identify apparent inequalities where none exist. While most of the commonly used disease cluster detection and disease modelling methods are useful for identifying general patterns and trends, few are designed to test explicit hypotheses about differences in absolute or relative risk from one region to another. Still, these same methods frequently report information that facilitates direct comparisons without adjusting for multiple testing. This problem has been well discussed in the statistical, epidemiology and surveillance literature, though few general purpose solutions to the problem exist [13-17].

### Modifiable areal unit problem

The modifiable areal unit problem describes the challenge of choosing geographic regions, areas or zones of representation in geographical analysis. Often choices are bound by practical issues--such as the data available. Other times, choices are limited by administrative realities--such as reporting health statistics for regional boards or other policy relevant jurisdictions. It is well known that many geographical analyses can produce very different results depending on the geography chosen for analysis. In the context of geographic public health surveillance, spatial anomalies may go undetected if anomalously high-risk places are organized into the same geographical group as anomalously low-risk places. The absence of an all-purpose solution to the modifiable areal unit problem means that careful consideration is often required to ensure that analysis is not greatly affected by decisions related to geographic grouping. In disease mapping applications, solutions to this problem include using multiple-membership models in which zones exert influence on their neighbours [8,9] thereby blurring the concept of a discrete geographic unit of analysis. Other research has sought to redistrict small areas into hierarchical systems in which smaller areas are enclosed or 'nested' into larger areas of [18-20] and therefore suitable for multi-level analysis and representation of health data. In both these cases, the choice of geography is not discrete in analytical terms, and to some degree, allows one to model some of the effects of the modifiable areal unit problem explicitly.

### Case ascertainment bias

When a gold standard case definition is unavailable or inappropriate, alternative case definition strategies are used. Perhaps most commonly these include using 'syndromic' definitions of infectious disease cases that lack laboratory confirmation [21] and multiple source case ascertainment algorithms for identifying chronic disease cases based on administrative health data [22,23]. Whatever the source of data, effective geographic public health surveillance requires that there are little or no systematic unexplained geographic differences in the ability to identify a person as a case. When such case ascertainment biases do exist, it obscures true differences in epidemiology. In the geographic public health surveillance, any tendency for a surveillance system to under-detect cases in certain places (e.g., rural areas) or in certain sub-populations (e.g., ethnic groups) makes it difficult to know whether apparent variations are due to variations in health, or variations in the effectiveness of identifying cases. Since case ascertainment bias may include underrepresenting disease in vulnerable sub-populations, it is both a methodological and ethical concern, particularly if the underrepresented populations are otherwise marginalized.

Importantly, these four challenges are not independent from one another. For example, the choice of geographic unit of analysis and representation will affect the statistical problems associated with small numbers; larger geographies are more statistically stable, but obscure higher resolution variations. Similarly, case ascertainment bias may be more apparent at some geographic resolutions than others, particularly if there are scale-specific factors that could influence case ascertainment--such as hospital catchment and health administration regionalization. While there is considerable literature describing and addressing each of these challenges independently, geographic health surveillance systems need to address these challenges together, or risk providing misleading information to decision makers and the public.

This paper has two objectives. First, we present a framework for geographic surveillance of chronic disease that integrates recent developments in case ascertainment methodology, data management and geographical analysis of disease to at least partly address the four challenges noted above. This framework is designed for use with population-based administrative health data, but is general enough to be applicable to many scales of analysis and different types of data. We propose this integrated framework with the specific hope of facilitating analysis of case ascertainment algorithms within a routine chronic disease surveillance system. Our second objective is to apply this framework to the geographic public health surveillance of asthma in Alberta, Canada, with a particular emphasis on the differences between Aboriginal and non-Aboriginal persons. This application is part of a national strategy to build capacity for surveillance of chronic diseases.

## Results

Here we describe the operation of the framework as applied in the province of Alberta, Canada. Alberta is a province in Western Canada that includes two cities of roughly 1 million inhabitants each, and a total population of over 3.5 million persons covering a geographic area more than twice the size of Poland. Alberta's economy is highly reliant on the export of oil and natural gas. In recent years, this has resulted in a substantial economic windfall, and lead to considerable migration into the province, both from other parts of Canada, and internationally. This has made the task of public health surveillance particularly important, since changing demands on the health care system and changing physical and social environments can have short and long term consequences on the health and well-being of the population.

### Definition of the data

Most permanent residents of Alberta are covered by the provincial health care system, and are included in an electronic public health insurance registry recording persons insured by the system. Each record in this registry system includes a unique person identifier, and other data maintained for administration purposes--such as address, date of birth and sex. The unique person identifier can be linked to a number of other electronic health data sources. We make use of three data sources to identify cases: a medical claims system that records services performed by physicians in the province, an inpatient hospitalization system that records inpatient admissions to hospitals and a hospital outpatient system that records admissions through outpatient services (such as the emergency department).

The linkage of information from the public health insurance registry to information about the use of health services has facilitated considerable work in population-based public health surveillance in Canada [22,24-26]. In most of these applications, International Classification of Diseases (versions 9 & 10) codes are crucial for identifying the illness associated with the health services. In most administrative data, these codes generally pertain to the health issue most responsible for a particular interaction (e.g., office visit) between patient and practitioner. Considerable work has gone into the evaluation of these codes as indicators of health status [23,27-29].

### Case ascertainment

This framework involves the identification of persons as cases based on their interaction with the health care system as captured by the three data sources mentions above. Other methods have been developed to combine information from multiple data sources for geographic surveillance [30]. Our approach uses a case ascertainment model in which multiple data sources with diagnostic information (in particular, International Classification of Disease (ICD) codes) are used to identify cases. This approach assumes that it is possible to meaningfully characterize illness status based on a mixture of administrative health data sources. This differs from the purely service-based case ascertainment approach common in syndromic surveillance, in which all (or generally less specifically defined) health-related contacts are of interest whether or not they correspond to a precise health condition.

While all three data sources are important for ensuring the most precise case ascertainment algorithm possible, the medical claims system is generally considered to contain considerable diagnostic noise and miscoding. However, it is also by far the largest source of information on the use of health care services, and cannot be excluded without resulting in a large drop in sensitivity. To deal with this problem, case ascertainment algorithms are usually adapted so that medical claims information is used only if there have been multiple medical contacts related to the illness of interest. Examples are two in two (2IN2) algorithms in which a person is a case only if they have had two or more claims-identified diagnoses for the condition of interest within a two year period [22]. Hospital inpatient and emergency department contacts in the medical system in Alberta are generally considered more reliable because of the higher quality of medical record keeping in hospitals, and because admission to hospital or emergency department generally coincides with greater clinical severity. For this reason, these data sources are less often restricted in the same way; for example, a single hospitalization or emergency department visit for asthma is generally considered sufficient evidence that the person has asthma. However, for other conditions, the framework can be adapted to require multiple emergency department contacts or hospitalizations, if such rules enhance the precision and accuracy of case ascertainment.

This framework allows easy selection of a specific case ascertainment algorithm so that it is possible to visualize how spatial patterns may change subject to different algorithms. For each case ascertainment algorithm, the framework also tabulates data on persons identified as possible cases that do not go on to meet the full case ascertainment criteria. We refer to these as 'residual' cases. For example, for the 2IN2 algorithm, the residual cases in a particular year are persons who had one medical claim associated with asthma, but no further medical claims associated with asthma in the year following and furthermore, did not meet the 2IN2 definition in any subsequent year. Similarly, for a 3IN2 algorithm, the residual cases are persons who had one or two medical claims associated with asthma in one year, but no further medical claims, and did not meet the 3IN2 definition in any subsequent year. Keeping track of patterns in the residual cases is useful for identifying case ascertainment biases as well as identifying unexpected changes or problems in the data system.

### Data structure

The public health insurance registry, medical claims, inpatient hospital and emergency department data sources are all linked together into a longitudinal panel. The panel includes counts of the number of asthma-related contacts with each of the three health data systems as well as annual snapshots of geographic location and demographic measures at the individual level. For this application, we use municipality of residence as our identifier of geographic location. A municipality includes cities, towns and other settlement areas as recognized by provincial legislation. Rural living people are assigned to the municipality where they pick up their mail.

For privacy and computational reasons, this panel is not directly useful for analysis, and so the panel data are organized into data cubes. Data cubes are reductions of flat-file tables into aggregate tables useful for storage and retrieval of large quantities of data. When there are repeat values in one or more of the attributes in a given data table, reduction (or aggregation), of these data into 'cubes' can reduce the size of the data structure without losing information. Typically, each attribute in the flat-file table is treated as a dimension, and each record or 'cell' within a cube contains summary statistics (such as counts, rates or standard errors). All individuals who have the exact same attribute values for all dimensions comprise the summary statistics in each cell. Table 1 illustrates a hypothetical table for all attributes used in this framework. In our application, the longitudinal panel has five dimensions: geographic location, age (in 10 year age group intervals), sex, status as Aboriginal (as defined by the Canadian constitution) and year. There are two summary measures recorded for each cell: the count of the number of cases (for a given case ascertainment algorithm) and population.

**Table 1:** Table of data cube structure (hypothetical counts)

Age Group	Sex	Aboriginal	Year	Municipality	COUNT cases (2IN2)	COUNT population
0 to 9	Male	No	2002	Edmonton	459	4230
0 to 9	Male	No	2002	Wetaskiwin	25	304
0 to 9	Male	No	2002	Vegreville	19	270
...	...	...	...	...	...	...
20 to 29	Female	Yes	2005	Airdrie	39	400
20 to 29	Female	Yes	2005	Canmore	32	327
20 to 29	Female	Yes	2005	Calgary	593	4899
...	...	...	...	...	...	...

**Figure 1:**
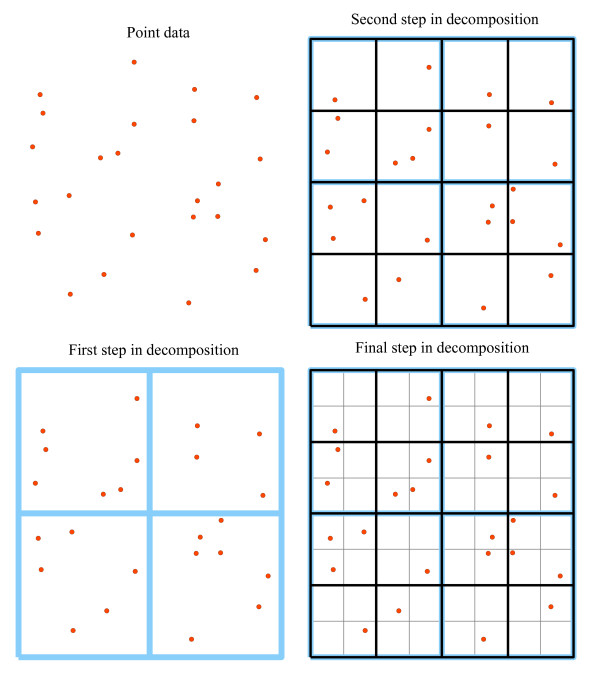
**Example of the decomposition of a plane of points into a quadtree tessellation of cells**.

Municipality of residence is the most precise geographic information contained in the panel (although more precise geographies are available in the population health insurance registry). In this application, each municipality is geo-coded to a point based on the population weighted centre of postal codes contained in the municipal area. These points are then spatially referenced to a spatial quadtree. In simple terms, a spatial quadtree is a nested spatial data structure in which geographical objects are subdivided into four 'child' geographical objects. In most applications, the quadtree structure is constructed through recursive decomposition of an area into progressively smaller units of quadrilateral polygons (Figure 1). In our application, starting at the highest level (the province), the region is split into four quadrilateral polygons, each of which is itself split into four quadrilateral polygons until no polygon includes more than one municipality. The result is an hierarchical tessellation in which each municipality is labelled as being enclosed by a polygon, which is itself included within a nested hierarchy of progressively larger polygons.

### Analysis

In this application, the lowest unit of analysis are cells in the data cube. Each cell in the data cube has a count of asthma cases (according to a particular case ascertainment algorithm) and population. The objective of the surveillance framework is to estimate absolute and relative risk associated with the cells in the cube. These estimates can then be used to construct maps (and other analytical information) for routine reporting. This is preferred over reporting crude rates or morbidity ratios for each of the cells in the cube, since an appropriate statistical model can be used to manage stochasticity in the data, as well as include known explanatory variables.

In geographic public health surveillance, the attribute of particular interest is geographic location. As noted above, the only explicit geographic attribute retained in the data is municipality of residence, which is organized into an hierarchical structure based on spatial quadtrees. This facilitates a multilevel approach to modelling disease patterns. We take a multilevel approach for three reasons. First, this approach allows us to model clustering at various levels of geography, thereby identifying the presence of geographic patterns at multiple scales. For decision makers, it may be useful to know at which scales a pattern is important, as it can inform the scale of intervention--for example, are the variations large regional trends, or small community-level variations? Second, incorporation of these random effects into our models serves to smooth out variations in asthma prevalence. This aids in the visualization and interpretation of patterns, particularly when the condition being reported is rare. Finally, this approach allows us to model random coefficient or slope effects, which can help identify whether or not the relationship between an explanatory variable (such as year) and prevalence of disease is constant over the whole study area, or if the relationship may systematically vary over space.

Count data can be modelled a variety of ways, and one of the most common is using Poisson regression. The Poisson regression model assumes the independence of observations and that the variance and mean of the response distribution are equal. A particularly important concern with respect to the data structure presented here is that there may be unexplained factors that could contribute to considerable heterogeneity in the estimates of risk between cells in the data cube. This is especially true for data cubes with a small number of dimensions, because we might expect that there are many unmeasured factors that could cause large unaccounted for variability in risk. In these cases, the assumption of equal mean and variance may not hold, and alternatives to the Poisson model will often yield better statistical inferences.

The negative binomial model is a generalization of the Poisson model in which an extra parameter is included in order to account for excess heterogeneity in the response distribution; this extra heterogeneity could be the result of misspecification (caused by missing model variables) or extra random variation between units of observation. The former is particularly relevant in the surveillance case, since the longitudinal nature of surveillance often prohibits collecting comprehensive data that create a more fully specified statistical model. The negative binomial model accounts for this extra heterogeneity by including a random effect *e*_*i *_that is assumed to have a gamma distribution with a mean and variance equal to *α*^-1^. As the value of this parameter approaches 0, the model is equivalent to the Poisson [31]. In most regression modelling applications, the negative binomial distribution function is linked to the log of the linear predictor, and like the Poisson, includes an offset term to account for inhomogenous distribution of population at risk.

The general structure of a single-level model used in this surveillance framework is

where the log of the mean *μ*_*i *_is a linear function of the fixed intercept *β*_0_, coefficients *β*_1 _through *β*_4 _associated with each of the predictor variables and *o*_*i*_, an offset term proportional to the population at risk. The term *e*_*i *_is the random effect meant to account for extra heterogeneity described above. In this multilevel approach, we also include random intercept effects for each level in the geographic hierarchy. Three levels of geography are used to identify clustering of prevalence at different spatial scales: the municipality level and two levels based on unique tessellations generated as part of the spatial quadtree. These areas are quadrilateral polygons of 156.25 and 2500 Km^2 ^in area; we refer to the former as 'small areas' and the latter as 'large areas'. The random intercepts associated with each of the three levels of geography are assumed to be normally distributed with means of 0 and variances of *υ*^2^, *τ*^2 ^and *φ*^2^. These three variance components indicate the degree to which there is clustering in the response variable at each level independent of variations at other levels, and independent of the fixed effects. Non-zero estimates of some and not other effects would indicate patterning or clustering at some scales and not at other scales. Finally, our model includes a random coefficient/slope effect to determine whether or not the relationship between Aboriginal status and risk of asthma is geographically homogenous.

### Visualization

The framework above can be used to estimate risk at the municipality level. Reporting results at this level is generally not informative because of the difficulty of visualizing broad trends in attributes represented by point data. Furthermore, the direct comparison of different municipal estimates facilitates the multiple comparisons problem that surveillance system developers often want to avoid. To address this issue, estimates are interpolated between locations of municipalities. Interpolation of point estimates into continuous surfaces of risk is frequently used to preserve confidentiality and to help reveal general patterns of disease [5]. We use an inverse distance weighted interpolation method to generate a map of continuous risk based on the modelled relative risk estimates associated with the municipalities, though other methods of spatial interpolation are equally suitable (e.g., spline smoothing and kriging). For this application of inverse distance weighted interpolation, we set the weighting coefficient to a small value (0.5) but placed a threshold of 150 kilometres to prevent municipalities at a very great distance from each other from having any effect on the interpolation process.

The framework, as presented here, is meant to integrate methods of data management, disease mapping and case ascertainment evaluation into a single system, and address some of the key challenges to routine geographic surveillance of chronic disease. The framework can be easily expanded to include a variety of commonly used methods in geographic surveillance--such as the spatial scan [32], regional cumulative sum (CUSUM) methods [33] and other spatial anomaly detection methods. Below, we describe an application of the framework to data on asthma prevalence.

### Asthma in Alberta: case ascertainment

**Figure 2:**
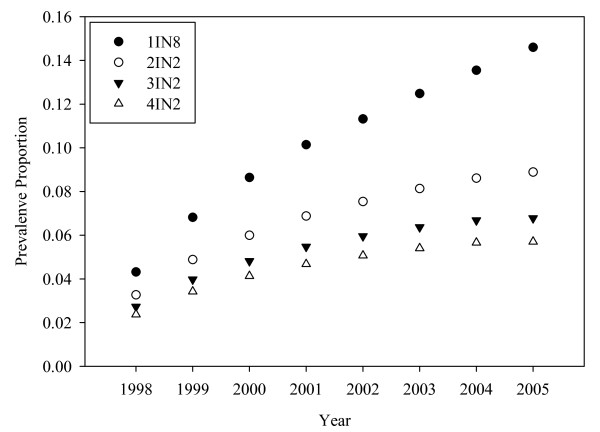
**Asthma prevalence proportion for different case ascertainment algorithms**.

There are differences in prevalence across the different case ascertainment algorithms, though the trends are similar (Figure 2). All algorithms here assume that hospitalizations or emergency department visits for asthma are sufficient to confirm a person as an asthma case. Other evidence suggests that in the case of asthma, case ascertainment is mostly influenced by the number of medical claims records than the absence or presence of hospitalization or emergency department visits [34]. The 1IN8 definition is the most sensitive available, since it considers any contact with the medical system for asthma over the surveillance period (1998 to 2005) as sufficient for the characterization of a person as asthmatic. As the first parameter of the algorithm gets larger (and more cases are required within a period of time to confirm a case) prevalence drops, though at an increasingly slower rate. Increases in the second parameter (the number of years within which cases are counted) has little effect on prevalence, and these results are not shown.

**Figure 3:**
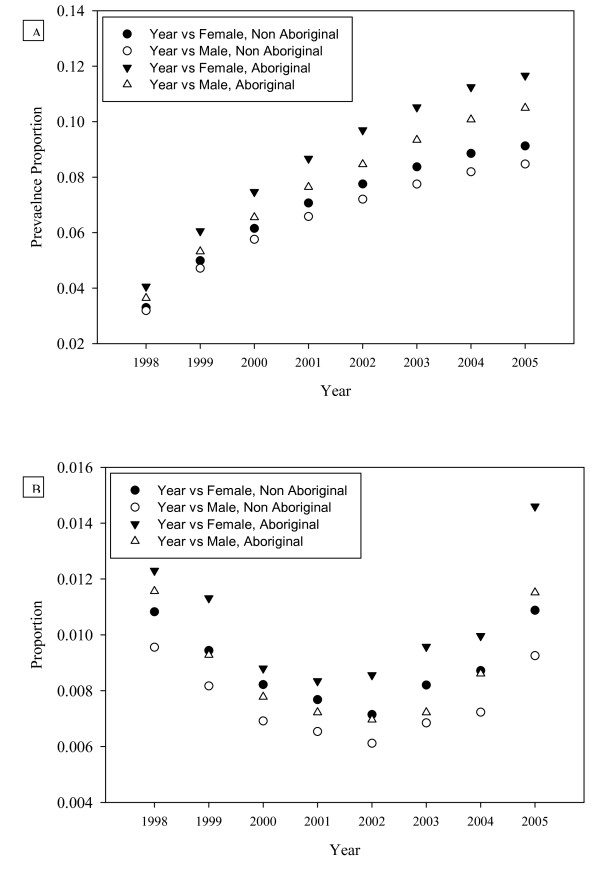
**A. Prevalence proportion of 2IN2 asthma by year, sex and status as Aboriginal**. B. Prevalence proportion of 2IN2 residual cases by year, sex and status as Aboriginal

The temporal pattern of residual cases differs from the distribution in prevalence (Figure 3A-B). In this figure, residual cases are those that qualify as cases according to the 1IN8 definition in a particular year, but do not qualify as cases according to the 2IN2 algorithm at any time between 1998 and 2005. While there are no apparent differences in the u-shaped pattern over time by gender or Aboriginal status, there are absolute differences by age and Aboriginal status, with female Aboriginals having the highest proportion of residual cases, and male non-Aboriginals having the lowest.

### Asthma in Alberta: multi-level models

The spatial quadtree structure allows many possible levels of geographic analysis, but we limit our analysis to the three levels described above. Our implementation solves this model using the SAS 9.2™ GLIMMIX procedure [35]. This procedure uses a doubly iterative linear approximation method that first fits a linear mixed model, and then uses the output from this model to update a linearization function, which generates new parameters for a new linear mixed model. This process is repeated until the convergence of parameter estimates. The use of pseudo-likelihood methods makes the comparison of traditional measures of goodness-of-fit (such as Akaike's information criteria) difficult. We therefore evaluated model fit by comparing the ratio of generalized chi-square statistic to the model degrees of freedom. Specifically, we compared the final negative binomial regression models in Tables 2 and 3 to equivalent Poisson regression models. For the model of asthma, this statistic suggests that the Poisson model is slightly over-dispersed (with a goodness-of-fit statistic greater than one) making the negative binomial model more appropriate. For the models of residual cases, there is less difference, suggesting that either the Poisson or negative binomial would have been appropriate.

**Table 2:** Model of 2IN2 asthma: 1998-2005, Province of Alberta, Canada

	Negative binomial			Poisson		
**Fixed effects**	**Estimate**	**SE**	**p-val**	**Estimate**	**SE**	**p-val**

Intercept	-31.7507	5.0709	< 0.0001	-32.7726	3.3903	< 0.0001
Female	0.0427	0.0045	< 0.0001	0.0906	0.0016	< 0.0001
Agegroup	-0.5253	0.0120	< 0.0001	-0.3805	0.0046	< 0.0001
Agegroup^2^	0.0568	0.0023	< 0.0001	0.0225	0.0009	< 0.0001
Aboriginal	12.8256	5.5017	0.0202	24.0478	3.4640	< 0.0001
Year	0.0163	0.0025	< 0.0001	0.0167	0.0017	< 0.0001
Year * Aboriginal	-0.0065	0.0027	0.0186	-0.0121	0.0017	< 0.0001

**Random effects**	**Estimate**	**SE**		**Estimate**	**SE**	

Large area (φ^2^)	0.0000	N/A		0.0000	N/A	
Small area (τ^2^)	0.0000	N/A		0.0000	N/A	
Municipality (υ^2^)	0.0236	0.0084		0.0390	0.0099	
Slope - Aboriginal	0.1624	0.0156		0.1617	0.0145	
Scale parameter	0.0493	0.0010		N/A	N/A	

Generalized Χ^2^/d.f.		0.89			1.93	

**Table 3:** Model of residual cases of asthma: 1998-2005, Province of Alberta, Canada

	Negative binomial			Poisson		
**Fixed effects**	**Estimate**	**SE**	**p-val**	**Estimate**	**SE**	**p-val**

Intercept	1.3819	0.0327	< 0.0001	1.3202	0.0232	< 0.0001
Female	0.1409	0.0079	< 0.0001	0.1679	0.0044	< 0.0001
Agegroup	-0.5916	0.0218	< 0.0001	-0.5257	0.0129	< 0.0001
Agegroup^2^	0.0721	0.0042	< 0.0001	0.0570	0.0025	< 0.0001
Aboriginal	-0.1121	0.0153	< 0.0001	-0.1053	0.0127	< 0.0001
Year	-0.2965	0.0078	< 0.0001	-0.2897	0.0043	< 0.0001
Year^2^	0.0329	0.0008	< 0.0001	0.0316	0.0005	< 0.0001

**Random effects**	**Estimate**	**SE**		**Estimate**	**SE**	

Large area (φ^2^)	0.0212	0.0105		0.0235	0.0090	
Small area (τ^2^)	0.0272	0.0085		0.0253	0.01003	
Municipality (υ^2^)	0.0025	0.0054		0.0029	0.0053	
Scale parameter	0.0349	0.0022		N/A	N/A	

Generalized Χ^2^/d.f.		0.98			1.06	

**Figure 4:**
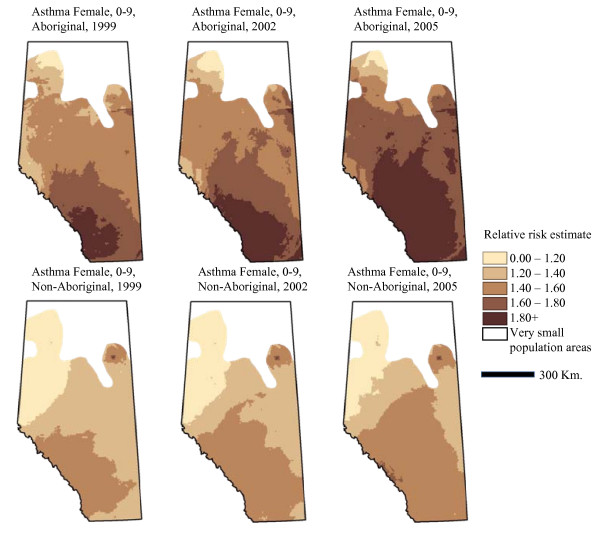
**Asthma relative risk in Aboriginal and non-Aboriginal Females, 0-9 years of age**.

Table 2 presents the fixed and random model parameter estimates for asthma prevalence in Alberta subject to the 2IN2 case definition criteria. The interaction between Aboriginal status and year is consistent with the apparent pattern in Figure 3A, in which the asthma prevalence seems to increase at a higher rate among Aboriginals than non-Aboriginals. The random intercept effects suggest that most of the variation occurs at small geographic scales--and in particular, between municipalities. The random slope effect associated with Aboriginal status (entered at the level of small areas) would suggest that the relationship between asthma risk and Aboriginal status varies in different regions of the province. We map the interpolated model estimates of relative risk in Figure 4. The maps illustrate patterns in relative risk of asthma by geography, time and Aboriginal status while holding sex and age constant. As expected, relative risk increases over time, and is higher among Aboriginal persons. The maps reveal that prevalence is highest in south-western Alberta, and lower in more northern regions, and particularly the north-west.

**Figure 5:**
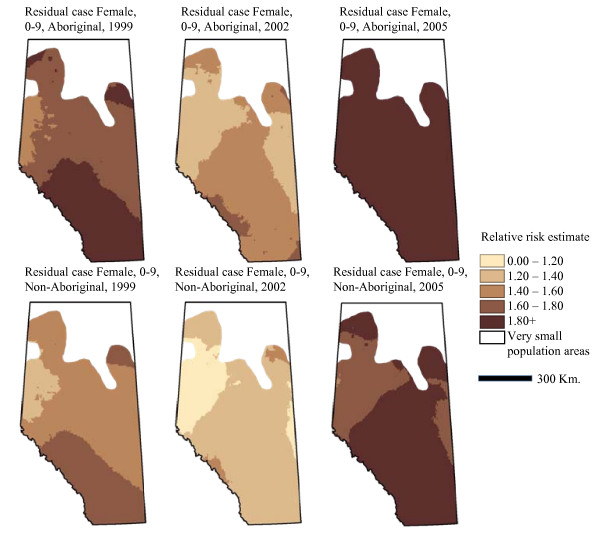
**Residual case relative risk in Aboriginal and non-Aboriginal Females, 0-9 years of age**.

Table 3 presents the fixed and random model parameter estimates for annual incidence of residual asthma cases as described above. While there are no fixed effect interactions included in this model, the non-linear trend on Figure 3B recommends including a quadratic term for year. In contrast to the model of asthma prevalence, the model of residual case incidence shows that most geographic variation occurs at larger geographic scales than the municipality-level. As above, we mapped relative risk of being a residual case while holding sex and age constant (Figure 5). The maps of residual asthma cases illustrate the non-linearity seen in Figure 3B, with risk apparently lowest in 2002. Similar to Figure 3B, this pattern may be explained by a number of factors, including variations in diagnostic methods or the effectiveness of the case ascertainment algorithms. The general geographic pattern of residual cases is very similar to the geographic patterns associated with asthma prevalence.

## Conclusion

Chronic disease surveillance differs significantly from most other public health surveillance activities. Chronic disease surveillance can be concerned with prevalence, severity and incidence, and must often take into account issues such as accessibility, improved diagnostic technology, improved treatment and other exogenous factors that can cause apparent trends in epidemiology. The syndromic surveillance model is effective for real-time surveillance of infectious disease outbreaks, because the cost of waiting for diagnostic precision is outweighed by the delay in public health response. Changes in chronic diseases usually occur over longer time frames, so the reporting delays resulting from increased diagnostic precision may often be acceptable. Administrative health data have proven useful sources of information for chronic disease surveillance in jurisdictions where these data are available, but when applied geographically, have often failed to take into account the mixture of methodological challenges unique to chronic disease surveillance.

We have presented a practical framework for the geographic surveillance of chronic diseases using administrative health data. The purpose of the framework is to address some of the key challenges of geographic public health surveillance in support of chronic disease surveillance generally. The multi-level approach helps to address the small numbers problem by smoothing out high-resolution variability with lower resolution random effects. Alternative disease mapping methods are available, but this approach has the added advantage of revealing the scales of spatial structure--for example, by distinguishing between local and/or regional trends. While this does not address the breadth of the modifiable areal unit problem, it does partly address the problem of scale, since variation in prevalence is being modelled explicitly at multiple scales simultaneously. The spatial interpolation of municipal-level estimates ensures that focus is on general patterns rather than specific estimates at specific geographic locations. This is particularly important in dissemination of information to policy makers, where the purpose of surveillance is to identify general trends and anomalies, rather than provide detailed estimates of prevalence at all locales. Finally, the analysis of residual disease prevalence facilitates the comparison of different case ascertainment algorithms, and ensures the framework is less likely to be overly influenced by any particular case ascertainment algorithm.

One of the purposes of public health surveillance is to generate potential questions for hypothesis driven health research. Several observations made above may warrant future investigation. First, our analysis of asthma prevalence suggests that while Aboriginal status is a risk factor for asthma, the relationship is not geographically homogenous; our analysis suggests that it could be stronger in some regions of the province, weaker in other areas, and non-existent in yet other areas. Future research could explain this effect as a function of an interaction between social and physical environments, or show that it is related to access to care. In Aboriginal populations where access to health care is poor, such as the rural north, true asthma sufferers may receive fewer diagnoses, and in turn, may be less often characterized as asthmatic by medical professionals. Urban living Aboriginals may be more likely to receive a diagnosis and treatment for asthma because of increased access to the medical system in general.

Second, we observed an unusual u-shaped pattern in the proportion of residual cases over time. The shape of the curve may reflect systematic changes in the use of medical services related to asthma, shifts in diagnostic behaviour over time (for example, changes in the tendency to diagnose bronchitis as asthma) or an artefact of the case ascertainment algorithm. The peak in 2005 in particular is at least partly due to the failure to identify all new cases in 2005; persons who will qualify in 2006 (with second diagnoses in the medical claims database) are missed in 2005, and appear as residual cases. However, the trend as a whole may reflect a systematic change in how asthma is diagnosed in the province. Future research should investigate which of the many possible explanations for this pattern is most likely, and then determine the effect of this pattern on routine surveillance activities. This observation in particular illustrates the importance of routine reporting and monitoring of case ascertainment algorithms over time.

Thirdly, we observed that the geography of residual cases is similar to the geography of 2IN2 cases--with high prevalence in south-western Alberta, and lower prevalence in the North. It is highly probable that residual cases include misdiagnosed instances of other respiratory illness (such as bronchitis). If true, our observations suggest that clinically similar respiratory conditions could exhibit a similar geography to asthma. This could point to an underlying social vulnerability to general respiratory illnesses in certain populations. This observation is also consistent with evidence that asthma can be caused and/or exacerbated by acute respiratory infections [36]. The similarity in patterns may also be an indicator of a practice style effect, such that practitioners in some regions have ways of characterizing respiratory illnesses that express geographical patterns independent of true variations in prevalence [37]. Untangling these possible explanations is warranted.

Finally, we observed that while geographic variation in asthma occurs at multiple scales, the model of asthma and model of residual cases of asthma have patterns that occur at different geographic scales. For the 2IN2 case definition model, most of the geographic variation occurs at higher resolutions--specifically, at the municipal level. This suggests that most of the geographic pattern in asthma is relatively localized, and concentrated in the variations between and within municipalities. In the model of residual disease cases, there is heterogeneity at all scales, but apparently more variation at lower resolutions than at higher resolutions. This is opposite to the pattern of asthma and suggests that most variation in the distribution of residual cases occurs regionally, rather than locally. Similar to our previous observation, this is intuitive if we consider the patterns in residual cases a proxy for patterns in physician practice style and or diagnostic error. Some practice style effects may result from professional interactions between physicians; within municipalities physicians may be more likely to share facilities and experiences and be influenced by common professional, administrative and regulatory forces. From this may emerge local practices of diagnosis that result in geographic clustering of patterns in residual cases. Such professional interaction and facility sharing may be harder to maintain at larger geographic scales, which would result in greater heterogeneity in style of practice from region to region.

In addition to prompting several possible research questions, our framework has some application to population-based public health practice. The spatial analysis of residual cases can be used to help identify geographical (and other) case ascertainment bias in syndromic and non-syndromic case definitions. This is important for assessing and enhancing the accuracy of surveillance information, including syndromic surveillance information, for which case ascertainment is usually intended to have high sensitivity to detect a signal, rather than precision in characterizing illness. Information on the distribution of residual cases can also be used to identify shortcomings in the health care system more generally. A cluster of residual cases in a particular region, or in a particular population, could suggest inequalities in the provision of diagnosis or treatment--such as the absence of adequate training or medical technology, or inequalities in opportunities for treatment. The analysis of the distribution of these residual cases may be an important secondary step in the public health surveillance process, over and above their uses as a tool for assessing case ascertainment.

Our analysis shows that Aboriginal Canadians have a disproportionate burden of asthma in Alberta, but that the magnitude of difference depends on the case ascertainment algorithm. This is not surprising since other research has shown that Aboriginal populations in Canada often have less access to the medical facilities and expertise necessary for asthma diagnosis [38,39]. This observation is consistent with a general critique of public health surveillance--routine chronic disease surveillance systems based on a single method of case ascertainment are likely to obscure differences between some population groups. This may be partly a problem of information inequity--information is often of lower quality in marginalized and economically disadvantaged communities [40]. Not only does this misrepresent patterns of disease, it misrepresents these patterns in a way that is likely to underestimate the true variation, and perhaps trivializes the true burden suffered by disadvantaged communities. Our results may recommend the development of sub-population specific health surveillance that takes into account the challenges of information inequity, particularly when there are significant variations in the distribution of residual cases. This could require the development of sub-population specific case ascertainment algorithms, rather than a single definition applied universally.

The framework we present is based on a source of administrative data common to most provinces in Canada, but that is not available in many other parts of the world. Our framework is of less applied value in settings where such information is unavailable. Unlike surveillance of acute health events or most notifiable infectious diseases, chronic disease surveillance in particular requires longitudinal sources of data. Changes in prevalence due to improved treatment or changes in epidemiology are difficult to monitor with cross-sectional data. While many jurisdictions have disease registries specific to particular outcomes (such as cancer and stroke registries), the bulk of chronic conditions cannot be effectively monitored without routinely collected population-based data. Therefore, while the framework we present is limited to application in regions where administrative data are available, the very task of comprehensive chronic disease surveillance is a challenge in regions where such data do not exist. We note that the availability of these data and the ability to conduct population-wide public health surveillance is one of the secondary benefits of having a single payer public health care insurance system.

## Competing interests

The authors declare that they have no competing interests.

## Authors' contributions

NY co-designed and implemented the framework, performed the analysis and wrote the manuscript. LS and DS co-designed the framework and edited the manuscript. All authors read and approved the final manuscript.
